# *Phellinus linteus* Grown on Germinated Brown Rice Inhibits IgE-Mediated Allergic Activity through the Suppression of Fc*ε*RI-Dependent Signaling Pathway *In Vitro* and *In Vivo*

**DOI:** 10.1155/2019/1485015

**Published:** 2019-11-29

**Authors:** Ha-Kyoung Kwon, Hye-Jin Park

**Affiliations:** Department of Food Science and Biotechnology, College of BioNano, Gachon University, 1342 Seongnan-daero, Sujeong-gu, Seongnam-si, Gyenoggi-do 461-701, Republic of Korea

## Abstract

*Phellinus linteus* (PL) has been used as a traditional herbal medicine owing to its immune regulatory activity. Previous studies reported that PL grown on germinated brown rice (PBR) exerted immunomodulatory, anticancer, and anti-inflammatory activities. However, role of PBR on type I hypersensitive reactions has not been studied yet. We found that PBR contained more polyphenolic compounds than PL extract. Among fractions, PBR butanol fraction (PBR-BuOH) significantly contained the most amounts of total polyphenolic contents compared with all extracts or fractions. In this study, anti-allergic activity of PBR-BuOH was examined using *in vitro* and *in vivo* models of immunoglobulin E/antigen- (IgE/Ag-) stimulated allergy. The inhibitory activity of degranulation was higher in PBR-BuOH (IC_50_ 41.31 ± 0.14 *μ*g/mL) than in PL-BuOH (IC_50_ 108.07 ± 8.98 *μ*g/mL). We observed that PBR-BuOH suppressed calcium influx and the level of TNF-*α* and IL-4 mRNA expression in a dose-dependent manner. The phosphorylation of Fyn, Gab2, PI3K, Syk, and I*κ*B protein is reduced by PBR-BuOH. Oral administration of PBR-BuOH inhibited allergic reactions including the extravasation of Evans blue dye, ear swelling, and infiltration of immune cells in mice with passive cutaneous anaphylaxis (PCA). These findings suggest that PBR-BuOH might be used as a functional food, a health supplement, or a drug for preventing type I hypersensitive allergic disease.

## 1. Introduction

Type I hypersensitivity reaction is mediated by immunoglobulin E (IgE) antibodies binding to high-affinity IgE receptors (Fc*ε*RI) [1–3] and includes allergic rhinitis, atopic dermatitis, and asthma [4, 5]. The prevalence rate of type I hypersensitivity is increased by triggers such as food, medications, insects, and unspecified causes over the last 10 years worldwide [6–11]. The current treatments for IgE-mediated allergic diseases are merely confined to the avoidance of allergens, anti-histamine treatment, and corticosteroid therapy with less efficacy and more side effects. However, it is reported that immediate hypersensitivity reactions could be induced by the unidentified food allergens [11–14], corticosteroids [11, 15], aspirin [16], progestogens [17], and antibiotics [18]. Current therapeutic agents for reducing the allergic reaction such as corticosteroids downregulated the expression of inflammatory mediators such as histamine and cytokines, but they can cause side effects that induce anaphylaxis involving urticaria, bronchospasm, angioedema, and cardiovascular collapse [11, 19]. Many reports are interested in improving the quality of care for those who suffer from allergic diseases [11] and examining whether the various complementary and alternative medicine therapies such as acupuncture, diet therapy, herbal medicines, and physical techniques are used for regulating diverse allergic disorders [7].

*Phellinus linteus* (PL) has been traditionally used as a natural medicine in Asian countries for immune regulatory activities [20–22]. Wild PL cannot be obtained in large quantities because they are difficult to grow on mulberry tree and are expensive [23]. In this study, we used *Phellinus linteus* grown on germinated brown rice (PBR), which is inoculated and cultured the mycelium of PL on germinated brown rice, that is, different from regular PL. In the previous studies, PBR exhibited physiological functions such as anti-inflammatory [24, 25], anticancer [26, 27], and anti-oxidant activities [23]. Unlike PL, PBR reduces IgE production through downregulating Th2 responses and has the immune-modulating of the balance of Th1 and Th2 cytokines in murine mesenteric lymph node lymphocytes [28]. The reducing IgE production of Th2 cell helps to modulate the hypersensitivity via suppressing IL-4 secretion and B cell activation in IgE-Fc*ε*RI-mediated allergic responses. In addition, previously they reported that ergosterol peroxide and atractylenolide III were found in PBR but not in wild PL [29, 30]. The contents of *β*-glucan and *γ*-aminobutyric acid in PBR was higher than that in regular PL and brown rice [25, 31]. Ergosterol peroxide possesses immunosuppressive [32], anti-inflammatory [33], and antibacterial effects [34]. *β*-glucan and *γ*-aminobutyric acid inhibited the allergic activity in both IgE-mediated mast cells and type I hypersensitivity animal models [35–37]. However, the effect of PBR and its underlying mechanisms against type I hypersensitivity reaction has not been studied yet. In this study, we investigated the inhibitory activity of PBR against IgE/Ag-stimulated RBL-2H3 cell activation and PCA reaction in mice.

## 2. Materials and Methods

### 2.1. Preparation of PBR Extract and Fractionation

**Figure 1:**
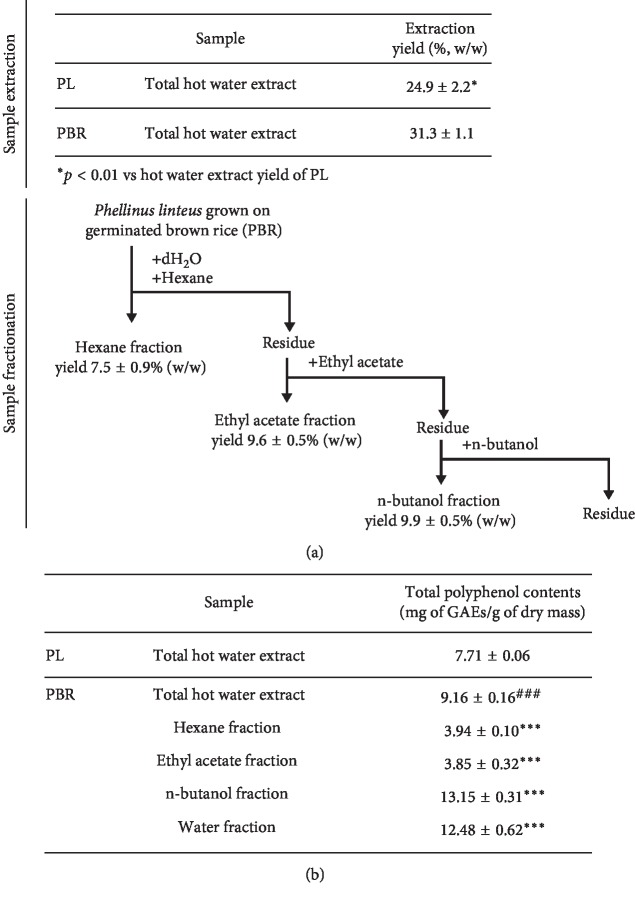
The procedure for sample extraction and fractionation (a) and the measurement of total polyphenol content in *Phellinus linteus* grown on germinated brown rice (PBR) and *Phellinus linteus* (PL) (b) (^###^*p* < 0.001 vs. total hot water extract of PL and ^*∗∗∗*^*p* < 0.001 vs. total hot water extract of PBR). Data are shown as mean ± SD values (*n*=3). GAEs, gallic acid equivalents.

Mycelia of PL (voucher number Kucari 0904) produced by solid-state culture and PBR (voucher number Kucari 0905) were kindly provided by the Cell Activation Research Institute (Seoul, Republic of Korea) and deposited in the Herbarium at the College of Bioscience and Biotechnology, Konkuk University (Seoul) [25, 26]. Each PBR or PL (1 kg) was powdered using a grinder and then was extracted with sterilized water (dry sample : water, 1 : 2) for 3 h at 95–100°C (Figure 1(a)). After filtration, the hot water extract was concentrated with a vacuum evaporator (Figure 1(a), yield of PBR and PL, 31.3 ± 1.1 and 24.9 ± 2.2%). Concentrated PBR was successively partitioned with hexane (yield 7.5 ± 0.9%), ethyl acetate (EA, 9.6 ± 0.5%), and water saturated n-butanol (BuOH, 9.9 ± 0.5%) (Figure 1(a)). PL also was successively partitioned with butanol using the same procedure as PBR. Each layer was concentrated by a rotary evaporator using vacuum (EYELA, Tokyo, Japan) at 40°C.

### 2.2. Determination of Total Polyphenol Contents (TPCs)

TPC in each extract was determined using a modified Folin–Ciocalteu method [38]. In each tube, 20 *μ*L of each extract and fractions of PBR or PL (50 mg/mL) and gallic acid (0–500 *μ*g/mL) was incubated with 20 *μ*L of Folin–Ciocalteu reagent for 5 min. Then, 120 *μ*L of 7% sodium carbonate (Na_2_CO_3_) was added and incubated at room temperature (RT) for 30 min. Absorbance was measured at 720 nm using a UV-Visible spectrophotometer (Epoch, BioTek Instruments, VT, USA). TPC was expressed as mg of gallic acid equivalents (GAEs)/g of dry mass.

### 2.3. Cell Culture

RBL-2H3 cells (rat basophilic leukemia cells, catalog number CRL-2256) were obtained from the American Type Culture Collection (ATCC) Biological Resource Center (Manassas, VA, USA). Cells were cultured in minimum essential medium (MEM; Invitrogen Co., Carlsbad, CA, USA) supplemented with 15% fetal bovine serum (FBS; Gibco, Grand Island, NY, USA) and 100 U/mL penicillin-streptomycin (Gibco) and were grown in a 75T cell culture flask at 37°C under humidified air containing 5% CO_2_ [1].

### 2.4. *β*-Hexosaminidase Secretion Assay

RBL-2H3 cells (2 × 10^5^ cells/well) were sensitized with 200 ng/mL of dinitrophenyl- (DNP-) specific IgE (Sigma-Aldrich, St. Louis, MO, USA) overnight. After washing with PIPES buffer (25 mM PIPES at pH 7.2, 119 mM NaCl, 5 mM KCl, 1 mM CaCl_2_, 0.4 mM MgCl_2_·6H_2_O, 40 mM NaOH, 5.6 mM glucose, and 0.1% BSA), cells were treated with PBR fractions (25 and 50 *μ*g/mL) or PP2 (Calbiochem, La Jolla, CA, USA), an Src tyrosine kinase inhibitor [1, 39]. PP2 blocks the phosphorylation of Syk [1, 39]. After 30 min, cells were stimulated with 200 ng/mL of antigen (DNP-BSA; Sigma-Aldrich, St. Louis, MO, USA) for 15 min at 37°C. Thereafter, the collected supernatant was mixed with 30 *μ*L of 1 mM p-nitrophenyl-acetyl-*β*-D-glucosaminide (p-NAG; Sigma-Aldrich) in 0.1 M citrate buffer (0.1 M sodium citrate, 0.1 M citric acid, pH 4.5) and incubated at 37°C for 2 h. Then, 200 *μ*L of 0.1 M Na_2_CO_3_/NaHCO_3_ solution (pH 10.0) was added to stop the reaction. Degranulation was calculated with the measurement of the released *β*-hexosaminidase as previously described [30, 40].

### 2.5. Fluorescence Assay of Intracellular Calcium Using Fluo-4 Direct Assay

Intracellular calcium levels were measured as previously described [30, 40, 41]. RBL-2H3 cells (2 × 10^4^ cells/well) were sensitized with 200 ng/mL of DNP-specific IgE overnight and then pretreated with PBR-BuOH (25 and 50 *μ*g/mL) for 30 min. RBL-2H3 cells were incubated with 50 *μ*L of Fluo-4 solution (Fluo-4 Direct Calcium kit, Invitrogen, Kumamoto, Japan) for 30 min at 37°C and then stimulated with 200 ng/mL DNP-BSA (Ag). Images were taken every 5 min on a fluorescent microscope (Nikon Eclipse Ti, Nikon Instruments, Melville, NY) using filter (excitation = 494 nm, emission = 516 nm). Then, the fluorescence intensity was quantitated by Image J software.

### 2.6. Cell Viability Assay

Cell viability was determined using the Cell Counting Kit-8 (CCK-8) (Dojindo Laboratories, Kumamoto, Japan) as described previously [1, 40]. RBL-2H3 cells (2 × 10^4^ cells/well) were incubated with PBR fractions or PL fraction (25 and 50 *μ*g/mL) at 37°C for 24 h, and then 10 *μ*L of CCK-8 solution was added and incubated at 37°C for 2 h. The absorbance was measured with a microplate reader at 450 nm (Epoch, BioTek Instruments, VT, USA).

### 2.7. Reverse Transcription-Polymerase Chain Reaction (RT-PCR)

Reverse transcription PCR was performed as described previously [1, 40]. Total RNA was isolated from RBL-2H3 cells, using TRIzol reagent, according to the manufacturer's protocol (Invitrogen, Carlsbad, CA). PCR was performed according to the manufacturer's protocol (Qiagen, Hilden, Germany). PCR program for TNF-*α* was done as follows: initial denaturation at 94°C for 2 min, followed by 30 cycles of denaturation at 94°C for 20 s, annealing at 62.2°C for 10 s, and extension at 72°C for 45 s, with a final extension at 72°C for 5 min. PCR program for IL-4 was done as follows: initial denaturation at 94°C for 2 min, followed by 30 cycles of denaturation at 94°C for 20 s, annealing at 56°C for 10 s, and extension at 72°C for 25 s, with a final extension at 72°C for 5 min. PCR program for GAPDH was done as follows: initial denaturation at 94°C for 2 min, followed by 30 cycles of denaturation at 94°C for 20 s, annealing at 62°C for 10 s, and extension at 72°C for 25 s, with a final extension at 72°C for 5 min [42]. PCR program for Fc*ε* receptor was performed as follows: initial denaturation at 95°C for 15 min, followed by 35 cycles of denaturation at 94°C for 30 s, annealing at 49°C for 90 s (for Fc*ε*RI *α*-subunit) or annealing at 51.9°C for 90 s (for Fc*ε*RI *β*-subunit), and extension at 72°C for 90 s, with a final extension step at 72°C for 5 min. PCR program for IFN-*γ* was done as follows: initial denaturation at 94°C for 2 min, followed by 30 cycles of denaturation at 94°C for 20 s, annealing at 57.3°C for 10 s, and extension at 72°C for 25 s, with a final extension at 72°C for 5 min. The following primers were used: TNF-*α* forward 5′-CAC CAC GCT CTT CTG TCT ACT GAA C-3′; TNF-*α* reverse 5′-CCG GAC TCC GTG ATG TCT AAG TAC T-3′; IL-4 forward 5′-ACC TTG CTG TCA CCC TGT TC-3′; IL-4 reverse 5′-TTG TGA GCG TGG ACT CAT TC-3′; Fc*ε*RI *α*-subunit forward 5′-AAT GGA TCC ACA ATG ATA GC- 3′; Fc*ε*RI *α*-subunit reverse 5′-AAT GAT GGG AAA ATG AGT TG-3′; Fc*ε*RI *β*-subunit forward 5′- GCA AAA GCT CTA CCA GAG AA-3′; Fc*ε*RI *β*-subunit reverse 5′-CTA CGC TCA AAT TCT TGT CC-3′; IFN-*γ* forward 5′-CA CAC TGC ATC TTG GCT TTG-3′; IFN-*γ* reverse 5′-TC CAC ATC TAT GCC ACT TGA G-3′; GAPDH forward 5′-CTT CAC CAC CAT GGA GAA GGC TG-3′; GAPDH reverse 5′-GAC CAC AGT CCA TGC CAT CAC TG-3′ (Cosmo Genetech, Seoul, Republic of Korea). The PCR product was separated by electrophoresis in 1.5% agarose gels. The bands were analyzed by RT-PCR using LI-COR Odyssey (LI-COR Biosciences lnc., Lincoln, NE, USA).

### 2.8. Western Blotting

Protein analysis was performed as described previously [1, 31, 40]. Cells (1 × 10^6^ cells/well) were lysed in RIPA cell lysis buffer, according to the manufacturer's protocol (Cell Signaling Technology, Beverly, MA, USA). The protein concentrations were determined using a BCA Protein Assay kit (Thermo Scientific, Rockford, USA). Equal amounts of proteins were loaded into each well and electrophoretically separated by 7–10% SDS-PAGE. The separated proteins were transferred to nitrocellulose membranes and blocked in 5% nonfat milk. Samples were probed with the following primary antibodies: phosphorylated Fyn (Santa Cruz, CA, USA), phosphorylated-the adaptor growth-factor-receptor-bound protein 2 (GRB2)-associated binding protein 2 (Gab2, Cell signaling technology, MA, USA), Gab2 (Cell signaling technology), phosphorylated-phosphoinositide 3-kinase (PI3K, Cell signaling technology), phosphorylated-Syk (Cell signaling technology), Syk (Cell signaling technology), phosphorylated-I*κ*B*α* (Cell signaling technology), NF*κ*B (Cell signaling technology), phosphorylated-ERK1/2 (Cell signaling technology), ERK1/2 (Cell signaling technology), and *β*-actin (Cell signaling technology). The membranes were washed in TBST (Tris Buffered Saline with Tween 20) buffer (Bio-Rad Laboratories, Hercules, CA, USA) and were incubated with horseradish peroxidase-labeled secondary antibody (Cell signaling technology or Santa Cruz). The bands were visualized and analyzed using LI-COR Odyssey (LI-COR Biosciences, Lincoln, NE, USA).

### 2.9. Passive Cutaneous Anaphylaxis (PCA)

Six-week-old female BALB/c mice, which are housed in under specific pathogen free conditions, were obtained from Orient Bio (Orient Bio Inc., Gyeonggi-do, Seongnam, Republic of Korea). They were maintained in cages at 22 ± 2°C and humidity 55 ± 5% and were exposed to 12 h light/12 h darkness cycles each day. Animals were randomly divided into four groups of six mice and were acclimated to laboratory conditions for 5–7 days. They were fed with standard diet and allowed free access to drinking water. All animal experiments were approved by the Institutional Animal Care and Use Committee (IACUC) at Gachon University (Orient Bio Inc., Gyeonggi-do, Seongnam, Republic of Korea) (GIACUC-R2017014).

DNP-specific IgE (1 *μ*g/mL) was injected into the ear of the mice. After 24 h, mice were intravenously injected with 1 mg/mL DNP-BSA (Ag) containing 1% Evans blue dye. To measure the activity of PBR-BuOH (25 mg/kg) or cetirizine (20 mg/kg, CZ), mice were orally administered the abovementioned dosages 1 h before DNP-BSA administration. The dye was extracted from each excised ear tissue with 700 *μ*L formamide at 63°C overnight as previously described [1, 30]. The absorbance was measured with a microplate spectrophotometer at 620 nm (Epoch, BioTek Instruments, VT, USA).

### 2.10. Histopathologic Assessment

Ear samples were fixed using 10% formalin and were embedded in paraffin as previously described [1, 43]. Paraffin-embedded sections were stained with hematoxylin and eosin (H&E); in general, the nuclei are stained blue-purple, whereas the cytoplasm and extracellular matrix are stained pink [1, 44]. Images of stained samples were taken by Nikon eclipse Ti (Nikon Instruments Incorporated, Melville, NY) using CCD camera (Point Grey Research Inc., Richmond, BC, Canada).

### 2.11. Statistical Analysis

Results are presented as means ± standard deviation (SD). Statistical differences between the control and the treatment groups were determined by Student's *t*-test and one-way ANOVA followed by Dunnett's *t*-test or Duncan's *t*-test (*p* < 0.05). The experimental data were analyzed using the Statistical Package for the Social Sciences-12 (SPSS Inc., Chicago, IL, USA).

## 3. Results

### 3.1. Total Polyphenol Content (TPC) in PBR

TPC in total hot water extract of PBR (9.16 ± 0.16 mg of gallic acid equivalents (GAEs)/g of dry mass, ^###^*p* < 0.001) was significantly higher than that in PL (7.71 ± 0.06 mg of GAEs/g of dry mass, Figure 1(b)). TPC was detected in all fractions (hexane, ethyl acetate, and n-butanol). Among the fractions, TPC in PBR butanol fraction (PBR-BuOH, 13.15 ± 0.31 mg of GAEs/g of dry mass of TPC, ^*∗∗∗*^*p* < 0.001) was highest among total water extract, hexane, or ethyl acetate fractions (Figure 1(b)). These data indicate PBR-BuOH might exert biological activity due to the presence of active molecules.

### 3.2. Effect of PBR on Degranulation of IgE/Antigen- (IgE/Ag-) Stimulated RBL-2H3 Cells

**Figure 2:**
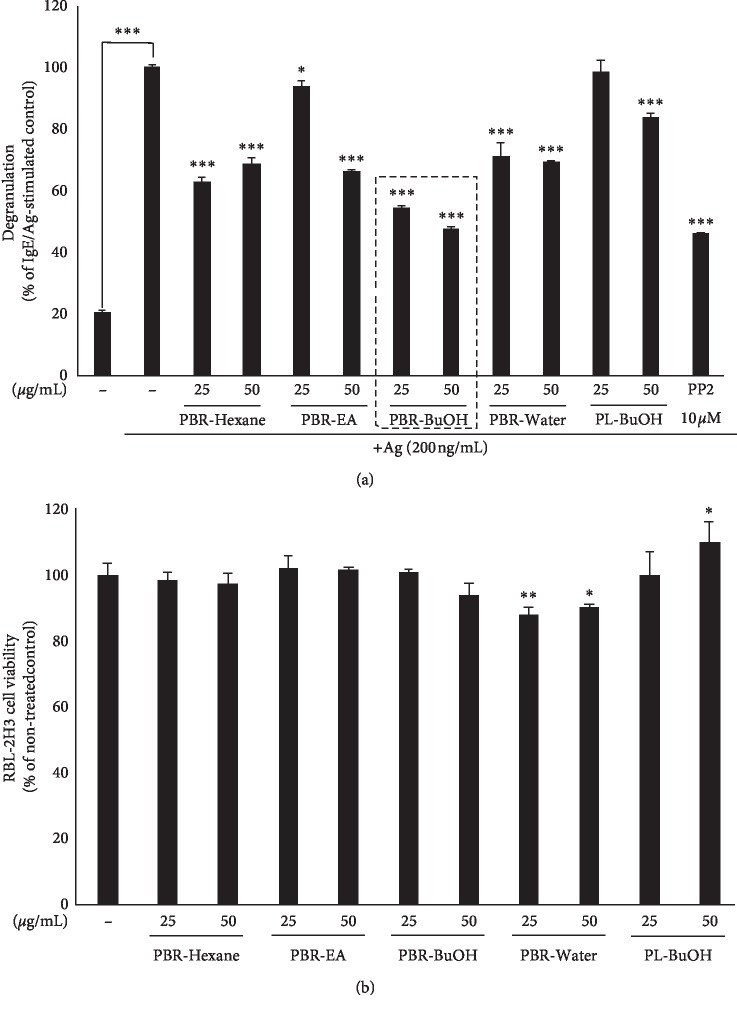
Effect of PBR fractions on degranulation of IgE/Ag-stimulated RBL-2H3 cells. (a) Degranulation was determined by the amount of *β*-hexosaminidase released in IgE/Ag-stimulated RBL-2H3 cells. RBL-2H3 cells were treated in the presence or absence of PBR or PL (^*∗∗∗*^*p* < 0.001, ^*∗*^*p* < 0.05 vs. IgE/Ag-stimulated control). (b) Cell viability of RBL-2H3 cells was measured using the Cell Counting Kit-8 (CCK-8) assay. RBL-2H3 cells (2 × 10^4^ cells/well) were treated in the presence or absence of PBR or PL fractions for 24 h. Each value represents the mean ± SD of three independent experiments. Data were analyzed with one-way ANOVA/Dunnett's *t*-test (^*∗∗*^*p* < 0.01, ^*∗*^*p* < 0.05 vs. nontreated control). EA, ethyl acetate; BuOH, butanol.

To elucidate the antiallergic activity of PBR (total hot water, hexane, EA, BuOH, and water), we evaluated whether they could inhibit *β*-hexosaminidase release (an index of degranulation) in IgE/Ag-stimulated RBL-2H3 cells. As shown in Figure 2(a), *β*-hexosaminidase release in the PBR-BuOH-treated group was inhibited at a concentration of 41.31 ± 0.14 *μ*g/mL (IC_50_, 50% inhibitory concentration of degranulation). IC_50_ values suggested that PBR-BuOH suppressed degranulation most efficiently compared to other PBR fractions. In addition, IC_50_ values of PBR-BuOH treatment (41.31 ± 0.14 *μ*g/mL) are lower than those of PL-BuOH treatment (108.07 ± 8.98 *μ*g/mL). Thus, our data showed that PBR-BuOH significantly inhibited degranulation in IgE/Ag-stimulated RBL-2H3 cells (Figure 2(a)) and had noneffective cell viability at a concentration of 25 and 50 *μ*g/mL (Figure 2(b)). Therefore, PBR-BuOH had been selected for further studies.

### 3.3. Effect of PBR on Proinflammatory Cytokines mRNA Expression in IgE/Ag-Stimulated RBL-2H3 Cells

**Figure 3:**
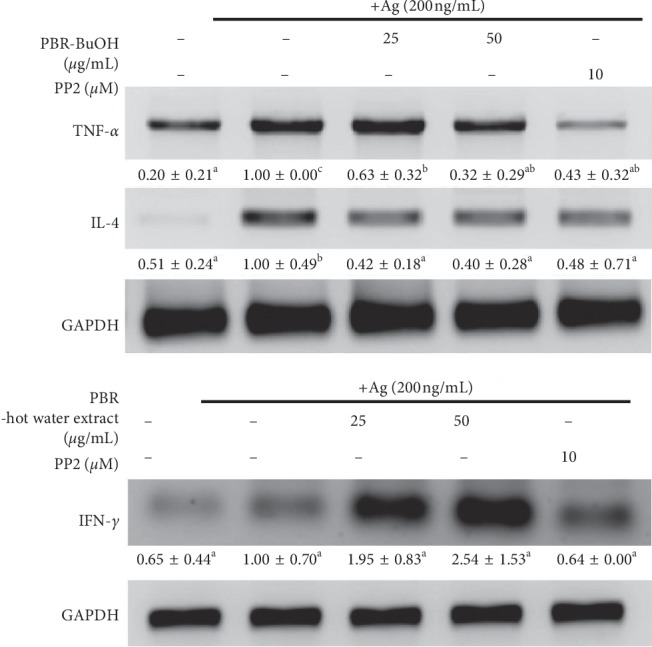
Effect of PBR on proinflammatory cytokines in IgE/Ag-stimulated RBL-2H3 cells. IgE-binding RBL-2H3 cells were stimulated with 200 ng/mL of DNP-BSA (Ag). Total RNA was isolated and then reverse-transcribed. The expression of TNF-*α*, IL-4, and IFN-*γ* mRNA were determined by quantitative RT-PCR. Representative images from three independent experiments are shown. Data were analyzed by one-way ANOVA/Duncan's *t*-test (*p* < 0.05). Different letters indicate significant differences between groups.

We evaluated whether PBR-BuOH decreased the level of IgE/Ag-induced TNF-*α* and IL-4 mRNA expression by RT-PCR in IgE/Ag-stimulated RBL-2H3 cells. IgE/Ag-stimulated RBL-2H3 secrete TNF-*α* and IL-4 that promote the release of inflammatory mediators such as nitric oxide, reactive oxygen species, and other cytokines and induce IgE antibody production of B cells compared with the nontreated control (Figure 3). PBR-BuOH (25 and 50 *μ*g/mL) significantly suppressed the level of IgE/Ag-induced TNF-*α* and IL-4 mRNA expression in a dose-dependent manner (Figure 3). In addition, PBR-hot water extracts significantly increased the level of IFN-*γ* mRNA expression in a dose-dependent manner in IgE/Ag-induced RBL-2H3 cells (Figure 3).

### 3.4. Effect of PBR on Calcium Influx of IgE/Ag-Stimulated RBL-2H3 Cells

**Figure 4:**
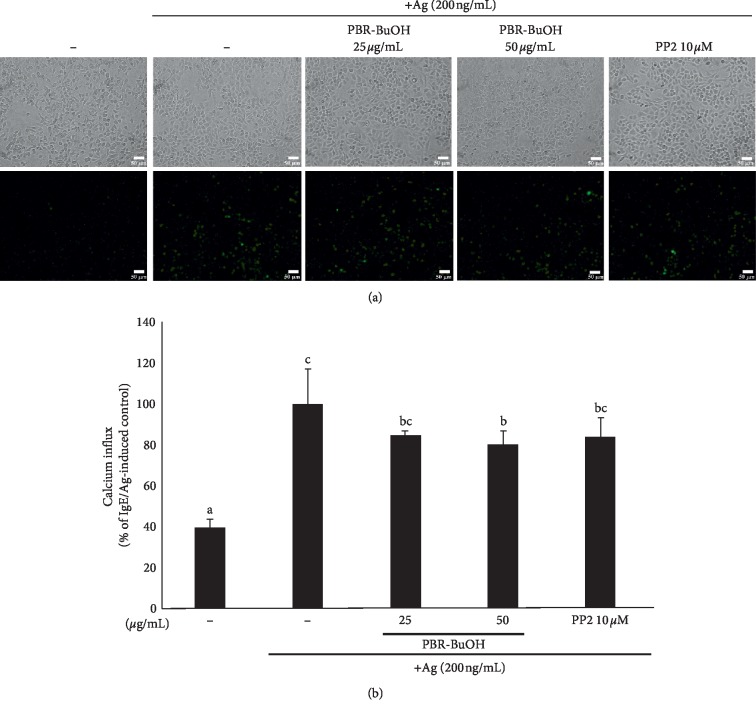
Effect of PBR-BuOH on calcium influx in IgE/Ag-stimulated RBL-2H3 cells. (a) Intracellular calcium levels were detected by Fluo-4 Direct Calcium Assay Kit. Scale bars indicate 50 *μ*m. (b) The fluorescence intensity was analyzed by Image J program. Each value represents the mean ± SD of three independent experiments. Data were analyzed by one-way ANOVA/Duncan's *t*-test (*p* < 0.05). Different letters indicate significant differences between groups.

IgE-mediated degranulation requires increased calcium mobilization [40, 41]. To evaluate whether PBR-BuOH affects the calcium influx induced by IgE/antigen, RBL-2H3 cells were stained with calcium-sensitive dye Fluo-4. We observed that fluorescence intensity decreased in PBR-BuOH-treated RBL-2H3 cells, compared with that in IgE/Ag-stimulated RBL-2H3 cells (Figure 4(a)). The quantitative analysis revealed that the levels of intracellular calcium were decreased in PBR-BuOH-treated RBL-2H3 cells (Figure 4(b)). This result indicated that PBR-BuOH can inhibit IgE/Ag-stimulated degranulation by suppressing the intracellular calcium mobilization.

### 3.5. Effect of PBR-BuOH on Fc*ε*RI mRNA Expression and the Activation of Fc*ε*RI-Dependent Signaling Molecules in IgE/Ag-Stimulated RBL-2H3 Cells

**Figure 5:**
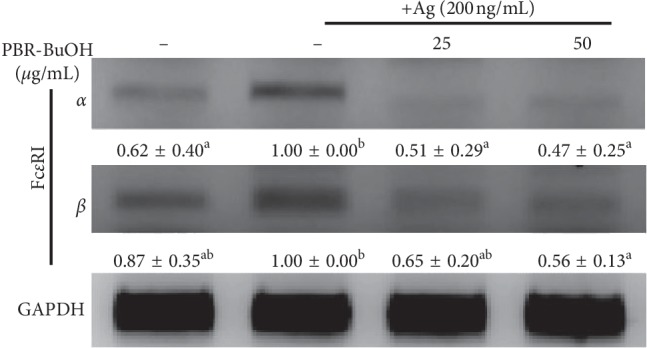
Effect of PBR-BuOH on Fc*ε*RI subunit mRNA expression in IgE/Ag-stimulated RBL-2H3 cells. IgE-binding RBL-2H3 cells were stimulated with 200 ng/mL of DNP-BSA (Ag). Total RNA was isolated and then reverse-transcribed. The expression of Fc*ε*RI subunit mRNA were determined by quantitative RT-PCR. Representative images from three independent experiments are shown. Data were analyzed by one-way ANOVA/Duncan's *t*-test (*p* < 0.05). Different letters indicate significant differences between groups.

To evaluate the level of IgE/Ag-induced Fc*ε*RI mRNA expression, we used by RT-PCR. PBR-BuOH (25 and 50 *μ*g/mL) significantly suppressed the level of IgE/Ag-induced Fc*ε*RI *α*-subunit and Fc*ε*RI *β*-subunit mRNA expression in a dose-dependent manner (Figure 5). These results indicated that PBR-BuOH might reduce the rate of Fc*ε*RI *α*-subunit cross linking with IgE and inhibit the activation of Fc*ε*RI *β*-subunit.

### 3.6. Effect of PBR-BuOH on the Activation of Fc*ε*RI-Dependent and NF-*κ*B Signaling Molecules in IgE/Ag-Stimulated RBL-2H3 Cells

**Figure 6:**
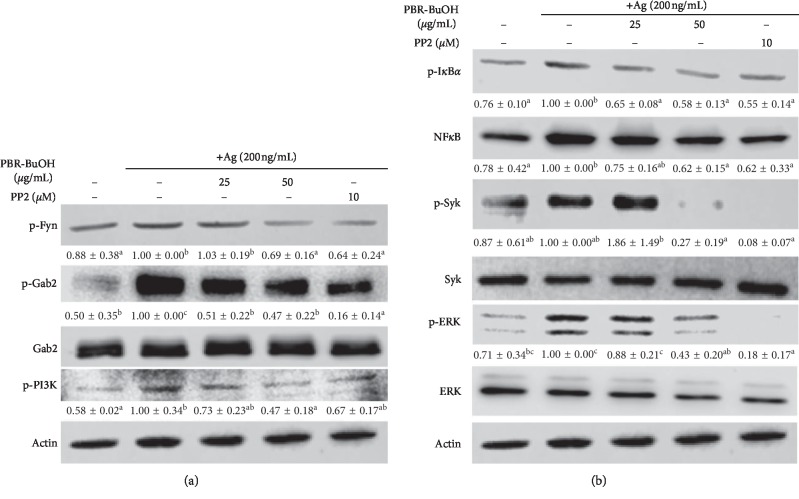
PBR-BuOH inhibited the activation of Fyn, Gab2, PI3K, I*κ*B*α*, NF*κ*B, Syk, and ERK proteins in IgE/Ag-stimulated RBL-2H3 cells. The levels of p-Fyn, p-Gab2, p-PI3K, p-I*κ*B*α*, NF*κ*B, p-Syk, and p-ERK proteins were measured by immunoblotting. PP2 is a general Src-family kinase inhibitor. Representative images from three independent experiments are shown as means ± SD and are analyzed by one-way ANOVA/Duncan's *t*-test (*p* < 0.05). Different letters indicate significant differences between groups.

**Figure 7:**
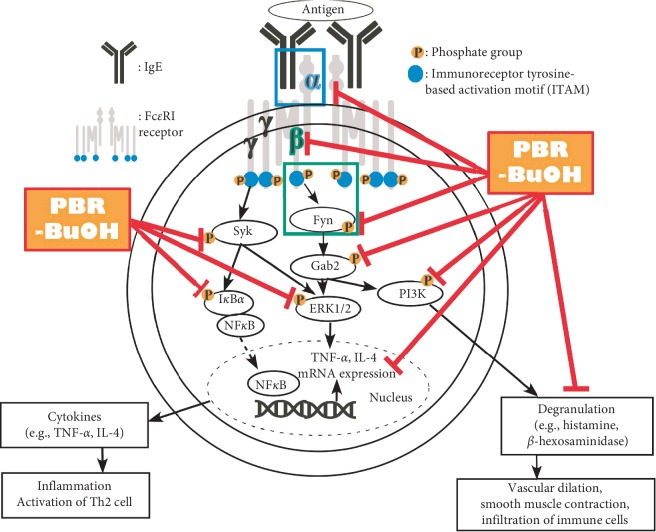
PBR-BuOH suppressed IgE/Ag-mediated allergic responses that are initiated by Fc*ε*RI signaling pathway. PBR-BuOH suppresses the level of Fc*ε*RI subunit mRNA expression and inhibits the activation of Fc*ε*RI-mediated signaling molecules, including Fyn and Syk. The level of p-I*κ*B*α* and NF*κ*B protein were inhibited by PBR-BuOH. PBR-BuOH also inhibited p-Gab, p-PI3K, and p-ERK which are other downstream signaling molecules. Red arrow indicates the inhibitory activity by PBR-BuOH through Fc*ε*RI-mediated and NF*κ*B signaling pathway in IgE/Ag-mediated allergic responses.

Activation of Fc*ε*RI signaling events in mast cells results in the degranulation and the production of proinflammatory cytokines. The Src family kinase (e.g., Fyn and Syk) and downstream molecules (e.g., Gab2, PI3K, and ERK1/2) are involved in Fc*ε*RI signaling pathways and NF-*κ*B signaling pathway. First, to elucidate the mechanism of its action, we investigated whether PBR-BuOH inhibited phosphorylation of Fyn, Gab2, and PI3K. PBR-BuOH decreased the expression of phosphorylated Fyn, Gab2, and PI3K in a dose-dependent manner compared with the IgE/Ag-stimulated control (Figure 6(a)). Here, we determined whether PBR-BuOH could suppress the activation of Syk, ERK1/2, NF-*κ*B, and I*κ*B*α*. PBR-BuOH decreased the levels of p-Syk, p-ERK1/2, NF-*κ*B, and p-I*κ*B*α* (Figure 6(b)). These results indicate that PBR-BuOH may exert its antiallergic activity through downregulation of the Fc*ε*RI-dependent and NF-*κ*B signaling pathway in IgE/Ag-stimulated RBL-2H3 cells (Figure 7).

### 3.7. Inhibitory Effect of PBR-BuOH on IgE/Ag-Mediated Passive Cutaneous Anaphylaxis (PCA) in BALB/C Mice

**Figure 8:**
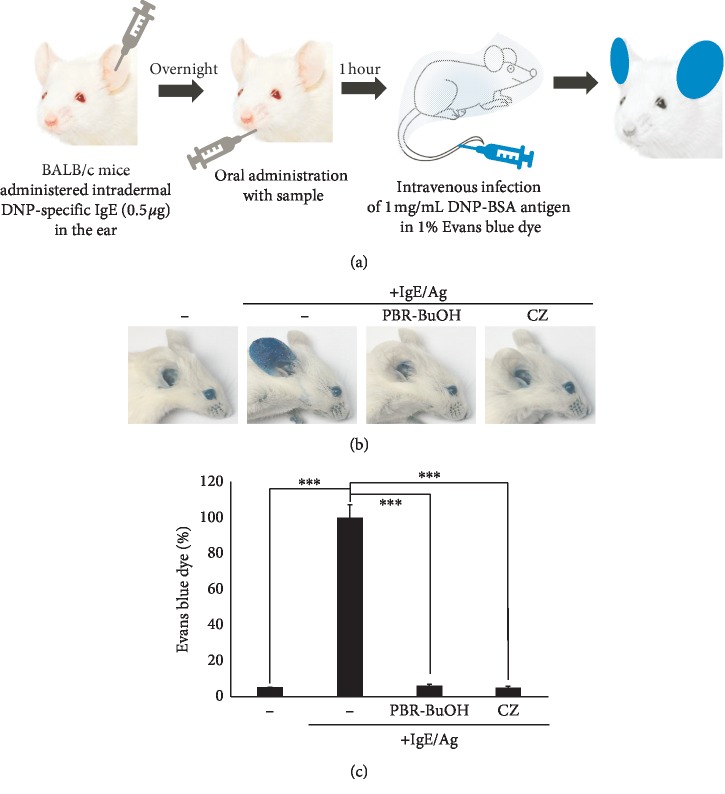
Effect of PBR-BuOH in BALB/c mice model on IgE/Ag-mediated PCA. (a) Scheme of the experimental design. DNP-IgE (1 *μ*g/mL) was injected into the ear of the mice. After 24 h PBR-BuOH or CZ was orally administered, and then DNP-BSA (1 mg/mL) containing 1% Evans blue dye was injected through the tail vein. (b) Representative images of ears after IgE/Ag-mediated allergic response. (c) The values of extravasated dye are expressed as % of IgE/Ag-stimulated control. Data were analyzed by one-way ANOVA/Dunnett's *t*-test (^*∗∗∗*^*p* < 0.001 vs. IgE/Ag-stimulated control).

To measure the inhibitory effect of PBR-BuOH on IgE/Ag-mediated type I allergic response, we used an IgE-dependent PCA that is immediate hypersensitivity reaction in the dermis. After IgE sensitization (injected into the ear), DNP-BSA (Ag) containing 1% Evans blue dye was injected through the tail vein (Figure 8(a)). PBR-BuOH, administered orally, at a dose of 25 mg/kg suppressed the amount of extravasated Evans blue dye in the ear of mice with IgE/Ag-mediated PCA, compared with IgE/Ag-stimulated control mice (Figures 8(b) and 8(c)).

### 3.8. Effect of PBR-BuOH on Histopathological Changes of Ear Tissues on IgE/Ag-Mediated PCA in BALB/C Mice

**Figure 9:**
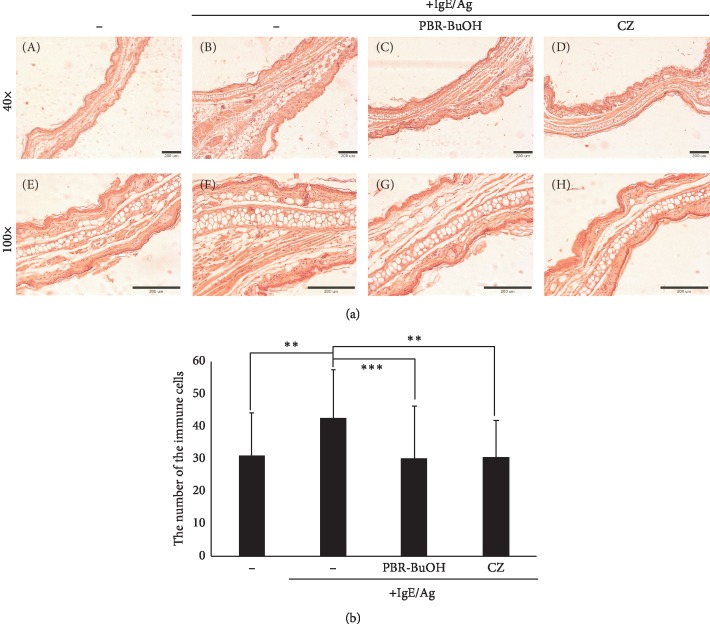
Histopathological analysis of permeated inflammatory cell and ear thickness by H&E staining of ear tissues in IgE/Ag-mediated PCA models. (a) The ear tissues were observed after H&E staining. (b) H&E stained tissues were measured to determine the changes in the number of immune cells after administering PBR-BuOH orally. Each value represents the mean ± SD of three independent experiments and was analyzed by one-way ANOVA/Dunnett's *t*-test. (*n* > 20; ^*∗∗∗*^*p* < 0.001, ^*∗∗*^*p* < 0.01 vs. IgE/Ag-stimulated control). Scale bars indicate 200 *μ*m. Each figure is representative of three independent experiments.

To identify inflammatory cell infiltration and ear swelling, the tissue sections were stained with H&E. The ear thickness in PBR-BuOH (25 mg/kg/day) or CZ (20 mg/kg/day)-treated mice was decreased, compared with that in the mice with IgE/Ag-mediated PCA (Figure 9(a)). The number of infiltrated immune cells was also decreased after the oral administration of PBR-BuOH (30.1 ± 16.0; *p* < 0.001) or CZ (30.3 ± 11.5; *p* < 0.05), compared with that in the IgE/Ag-stimulated control group (42.4 ± 14.9) (Figure 9(b)). These results suggest that PBR-BuOH suppressed the infiltration of inflammatory cells and reduced dermal and epidermal thickening, compared with that in the IgE/Ag-stimulated control group.

## 4. Discussion

In the present study, we demonstrated that *Phellinus linteus* grown on germinated brown rice (PBR) alleviated IgE/Ag-stimulated hypersensitivity by suppressing Fc*ε*RI-mediated pathway along with improvements in antiallergic activity. Recently, several studies reported that PBR showed enhanced biological activities, including immune regulation [28], anti-inflammatory [24, 25], anticancer [26, 27], and antioxidant activities, compared with PL [23]. The levels of ergosterol peroxide and atractylenolide III was only found in PBR, not in PL [25, 29]. It is reported that ergosterol peroxide has anticancer activity against colon cancer cells [33] and atractylenolide III has antiallergic activity by inhibiting IgE/Ag-mediated hypersensitivity response [30]. Other groups reported that both PL and germinated brown rice exert anti-inflammatory and antiallergic activities [24, 25]. In addition, PL or germinated brown rice contains the polyphenolic compounds such as protocatechuic acid and gallic acid, which inhibited the expression of cytokines (TNF-*α* and IL-4) exerted by IgE-mediated allergy-induced basophils [45–48]. In this study, we found that the total polyphenolic content (TPC) in hot water extract of PBR was significantly higher than that in hot water extract of PL. Particularly, PBR-BuOH contained the highest amount of TPC compared with the other fractions (Figure 1). However, the antiallergic activity and its mechanism of PBR have not been reported yet. Thus, in this study, we investigated whether PBR-BuOH showed the antiallergic activity and its mechanisms in IgE/Ag-mediated allergy responses.

To identify the antiallergic effect of PBR-BuOH, we observed the released inflammatory mediators of degranulation in IgE/Ag-stimulated RBL-2H3 cells. The granules of basophils and mast cells are degranulated by IgE-mediated reactions, cross linking with Fc*ε*RI, and IgE/antigen (Ag)-complex [1, 40, 49]. Activated basophil initiates the degranulation of secretory granules including histamine, serotonin, and *β*-hexosaminidase [50]. Among them, *β*-hexosaminidase is one of the lysosomal enzymes, which may be fused with secretory lysosome and endosome in RBL-2H3 cells [51]. It is used as an indicator of degranulation [52–54] and has defense against bacterial infiltration by degrading the cell wall peptidoglycan [55]. In the present study, PBR-BuOH markedly reduced degranulation, suppressing the activity of released *β*-hexosaminidase in IgE/Ag-stimulated RBL-2H3 cells (Figure 2). PBR-BuOH also exhibits enhanced degranulation-suppressing activity compared with PL-BuOH (Figure 2). As the immediate hypersensitivity continues, a large amount of cytokines (IL-3, IL-4, IL-5, IL-6, TNF-*α*, and GM-CSF) and chemokines (IL-8, and MIP1*α*) are secreted by IgE/Ag-stimulated basophils and mast cells within a few hours [50, 51]. Some cytokines such as TNF-*α* and IL-4 can be used as fascinating therapeutic target molecules for IgE-mediated allergic responses [51, 54, 56] and proinflammatory Th2 cell responses in allergic diseases [57]. TNF-*α* is found in secretory granules and induces endothelial activation, adhesion molecule expression, and inflammatory cell recruitment [52]. IL-4 acts upon naive T-cells, induces them to differentiate into allergen-specific Th2 cells, and stimulates B-cell differentiation to produce IgE [50, 58–60]. Some studies conducted on allergic rhinitis and asthma showed that there is an association of IL-4 with significant increase in TNF-*α* [56]. Previous reports have shown that PBR inhibits the production of IgE by modulating the Th1/Th2 balance, decreasing the concentration of IL-4, a Th2 cytokine, in murine lymphocytes [28]. Therefore, we examined whether PBR-BuOH suppressed IgE/Ag-induced gene expression of TNF-*α* and IL-4. We found that PBR-BuOH significantly reduced TNF-*α* and IL-4 mRNA expression (*p* < 0.05, Figure 3). In addition, we found that PBR-hot water extract increased the level of IFN-*γ* mRNA expression in RBL-2H3 cells (Figure 3), which enhances Th1-mediated responses and inhibits Th2 differentiation and mast cell activation [61–64]. This result suggests that the antiallergic effect of PBR-BuOH is a result of its reduction of TNF-*α* and IL-4 expression in basophils.

Basophils and mast cells have Fc*ε*RI for IgE on their cell membranes [65, 66], which is divided into the IgE-binding portion (*α*-subunit) and the signaling portion (*β*- and *γ*-subunits). Some asthmatic patients have more Fc*ε*RI-expressed immune cells, compared with non-asthmatic control patients [67, 68]. Increased numbers of Fc*ε*RI-expressed immune cells can rapidly respond to small amounts of IgE/antigen (Ag) complex and lead to the allergic reaction by releasing cytokines, chemokines, and other mediators, leading to activation of recruited other immune cells [69]. In brief, reducing the number of immune cells that can bind the IgE/Ag complex is targeted for treating hypersensitivity reaction. Some studies have also reported that Fc*ε*RI *α*-subunit-deficient mice do not induce degranulation because IgE cannot bind to the cell membrane receptor of basophils and mast cells [1, 70]. Since IgE-binding Fc*ε*RI *α*-subunit activates the antigen-binding site of IgE molecule, induction of Fc*ε*RI *α*-subunit increases IgE-mediated allergy [59, 71–73]. Our data showed that PBR-BuOH decreased the expression of Fc*ε*RI *α*-subunit mRNA in IgE/antigen-stimulated RBL-2H3 cells (Figure 5). Therefore, PBR-BuOH mitigates the allergic response by inhibiting the expression of Fc*ε*RI *α*-subunit, which it induces the degranulation of granules.

The IgE/Ag-mediated degranulation process can be divided into two phases: (1) the translocation of granules to cell membrane [74] and (2) the fusion between granules and cell membrane [53, 74, 75]. First, Fyn/Gab2/PI3K signaling pathway was activated by the phosphorylation of Fc*ε*RI [52, 65, 76–79]. Fyn and Gab2 can regulate not only the formation of microtubule but also the translocation of granules to the cell membrane [52, 74, 78, 80]. In this study, we found that PBR-BuOH significantly decreased the expression of Fc*ε*RI *β*-subunit mRNA in IgE/Ag-stimulated RBL-2H3 cells (Figure 5) and significantly inhibited the phosphorylation of Fyn, Gab2, and PI3K (Figure 6(a)). Our observations suggest that PBR-BuOH might inhibit the translocation of granules for degranulation by negatively regulating Fc*ε*RI-mediated signaling pathway. Second, we postulate that PBR-BuOH would suppress the calcium-dependent exocytosis of cytoplasmic granules. The increased intracellular calcium constitutes calcium-calmodulin complex that leads to the exocytosis of granules by inducing the SNARE complex activation [53, 74, 75]. Consistent with previously reported studies, we found that stimulation of IgE/Ag increases the levels of intracellular calcium in RBL-2H3 cells (Figure 4) [40, 41]. We observed that PBR-BuOH inhibited calcium influx assay compared with that in IgE/Ag-stimulated RBL-2H3 cells (Figure 4). We have deduced the reason for blocking degranulation by PBR its suppressing activity on the activation of Fc*ε*RI-mediated signaling molecules and calcium influx. Additionally, NF*κ*B signaling pathway induces the production of inflammatory cytokines in IgE-mediated allergic reaction [81–83]. Some studies demonstrated that phosphorylated Syk is known to induce NF*κ*B and NFAT activation [84]. As shown Figure 6, PBR-BuOH reduced total NF*κ*B protein expression and inhibited the phosphorylation of I*κ*B*α*, Syk, and ERK1/2 MAP kinase protein in IgE/Ag-stimulated RBL-2H3 cells (Figure 6(b)). These data suggest that PBR-BuOH possesses antiallergic activity by suppressing the level of phosphorylated Syk, ERK1/2 kinase protein, and I*κ*B*α* protein.

PCA is a representative local type I allergy that is induced in mice by injecting IgE into the ears and intravenously injecting antigen into the tails [30, 85, 86]. The antigen-antibody interactions increase the production of vasoactive substances such as histamine in immune cells of subcutaneous tissue [87] and induce the permeability and extravasation of the Evans blue dye injected into the circulation along with the antigen [88]. We observed that PBR-BuOH decreased the amount of extravasated Evans blue dye in the ear of mice, compared with that in the IgE/Ag-mediated allergic group (Figure 8). IgE-mediated PCA induces the expression of endothelial selectins and trafficking molecules that can bind with immune cells [89], and then the infiltration of immune cells occurs in the ear tissues [90–94]. The results showed that PBR-BuOH significantly inhibits IgE-mediated PCA by reducing the number of infiltrating inflammatory cells and the ear swelling (Figure 9). In this study, these findings implied that PBR-BuOH decreased the vascular expansibility and the activated immune cells caused by the degranulation and released cytokines of IgE/Ag-stimulated basophils and mast cells in dermis, which were in accordance with our *in vitro* results. In addition, Hong et al. conducted a clinical trial in which PBR powder was orally administered for 12 weeks to an atopic dermatitis patient at an average age of 7.3 years [95]. The symptomatic effect of atopic dermatitis, a type of hypersensitive reaction, was verified based on previous research that has significantly decreased the itching and objective SCORAD indices of the patient [95].

## 5. Conclusion

Our study demonstrated that PBR-BuOH has more potent antiallergy activity than PL-BuOH in IgE/Ag-mediated allergic response through suppressing degranulation and decreasing the level of TNF-*α* and IL-4 mRNA expression. PBR inhibited the phosphorylation of Fyn, Gab2, and PI3K in Fc*ε*RI-mediated signaling pathway and calcium mobilization. In addition, PBR-BuOH suppressed IgE-mediated type I hypersensitivity in the PCA murine model. Our findings suggest that PBR could be developed as functional food, health supplement, and drug for treating or preventing type I allergic diseases.

## Data Availability

*Phellinus linteus* grown on germinated brown rice (PBR, Kucari 0905, Patent: 1280949) and the mycelium of *Phellinus linteus* (PL, Kucari 0904, PDK4708) used to support the findings of this study have been deposited in the Cell Activation Research Institute Co., Ltd. The data used to support the findings of this study are currently under embargo while the research findings are commercialized. Requests for data, 6 months after publication of this article, will be considered by the corresponding author.
